# PlanHab Study: Consequences of combined normobaric hypoxia and bed rest on adenosine kinetics

**DOI:** 10.1038/s41598-018-20045-5

**Published:** 2018-01-29

**Authors:** C. Strewe, R. Zeller, M. Feuerecker, M. Hoerl, S. Matzel, I. Kumprej, A. Crispin, B. Johannes, T. Debevec, I. B. Mekjavic, O. Eiken, M. Thiel, G. Schelling, A. Choukèr

**Affiliations:** 1 Department of Anaesthesiology, University Hospital, LMU Munich, Laboratory of Translational Research “Stress and Immunity”, Munich, Germany; 2 Institute for Medical Information Processing, Biometry and Epidemiology, Klinikum Großhadern, University of Munich, Munich, Germany; 3 Division of Space Physiology, Institute of Aerospace Medicine, German Aerospace Center (DLR), Cologne, Germany; 4 Department of Automation, Biocybernetics and Robotics, Jozef Stefan Institute, Ljubljana, Slovenia; 5 Department of Environmental Physiology, Swedish Aerospace Physiology Center, School of Technology and Health, Royal Institute of Technology, Stockholm, Sweden; 6 Department of Anaesthesiology and Surgical Intensive Care Medicine, University Medical Center Mannheim, Medical Faculty Mannheim, University of Heidelberg, Heidelberg, Germany; 7 Department of Biomedical Physiology and Kinesiology, Simon Fraser University, Burnaby, British Columbia Canada; 8 Faculty of Sport, University of Ljubljana, Ljubljana, Slovenia

## Abstract

Adenosine plays a role in the energy supply of cells and provokes differential, hormone-like functions in circulating cells and various tissues. Its release is importantly regulated by oxygen tension. This renders adenosine and its kinetics interesting to investigate in humans subjected to low oxygen conditions. Especially for space exploration scenarios, hypoxic conditions – together with reduced gravity - represent two foreseen living conditions when planning manned long-duration space missions or planetary habitats. The PlanHab study investigated microgravity through inactivity in bed rest and normobaric hypoxia to examine their independent or combined effect on adenosine and its kinetics. Healthy male subjects (n = 14) completed three 21-day interventions: *h*ypoxic *b*ed *r*est (HBR); *h*ypoxic *amb*ulatory confinement (HAMB); *n*ormoxic *b*ed *r*est (NBR). The interventions were separated by 4 months. Our hypothesis of a hypoxia-triggered increase in adenosine was confirmed in HAMB but unexpectedly also in NBR. However, the highest adenosine levels were noted following HBR. Furthermore, the percentage of hemolysis was elevated in HBR whereas endothelial integrity markers stayed low in all three interventions. In summary, these data suggest that neocytolysis accounts for these effects while we could reduce evidence for microcirculatory changes.

## Introduction

The endogenous nucleosid adenosine exists ubiquitously in the intra- and extracellular space of the different organ tissues. Extracellular adenosine is generated predominantly through phosphohydrolysis of 5′-adenosine tri-/monophosphate (ATP/AMP).

Over the last years it has been demonstrated that adenosine influences and modulates multiple different physiological processes^1,2^ and that hypoxia plays an important regulatory role^3–6^. Adenosine acts through four G protein-coupled adenosine receptors that either stimulate (A_2A_, A_2B_) or inhibit (A_1_, A_3_) adenylate cyclase and thus enhance or decrease the second messenger cyclic AMP (cAMP). Finally, extracellular adenosine is rapidly taken up into the cells and metabolized either into inosine by adenosine deaminase or into AMP by adenosine kinase. As hypoxia does not allow sufficient and oxygen dependent re-phosphorylation of ADP and AMP, adenosine concentrations could increase as a function of hypoxia. Environmental hypoxia is not only a natural condition in higher altitudes or in aviation, but is also strongly considered as an atmospheric condition for future manned missions to outer space. Therefore, research on the effects of hypoxic environmental and living conditions in addition to reigning microgravity is of emerging interest for planning possible planetary habitats on the moon or Mars, or for long-duration space exploration class missions. In light of these scientific questions, the Planetary Habitat Simulation Study (PlanHab) was performed. We investigated the changes in adenosine release and its kinetics under simulated space conditions by subjecting 14 male subjects to 21 days of hypoxia and/or horizontal bed rest to mimic microgravity effects. We specifically investigated the course of the adenosine release, its concentrations and that of its metabolite inosine and were further interested, if and to what degree the adaptation to these standardized living conditions are linked to adenosine profiles and different physiologic states. We hypothesized that adenosine concentrations would be enhanced during the early stages of both hypoxic interventions. Our unexpected additional finding of hypoxia-independent adenosine increase has initiated further analysis to i) identify a potential role of hemolysis as a source of adenosine in our experimental interventions, ii) to evaluate the interaction of adenosine and endothelial barrier function, and iii) to investigate the impact of physical activity on adenosine concentrations.

## Material and Methods

The Planetary Habitat Simulation Study (PlanHab) was an EU-funded research program (Call FP7-SPACE-2011-1; project number 284438) ministered by several research groups investigating physiological and psychological effects of life in simulated planetary habitat conditions. The study protocol has been registered at ClinicalTrials.gov with the identifier NCT02637921 on December 1^st^, 2015. It was approved by the Committee for Medical Ethics at the Ministry for Health of the Republic of Slovenia. The Declaration of Helsinki was respected and bed rest protocols met the criteria of the European Space Agency (Standardization of bed rest study conditions, Version1.5, August 2009; revised on an international basis in 2014 in the ‘Guidelines for Standardization of Bed Rest Studies in the Spaceflight Context’ by the International Academy of Astronautics (IAA) (www.nasa.gov/sites/default/files/atoms/files/bed_rest_studies_complete.pdf)). All methods used were performed in accordance to standard laboratory guidelines and regulations.

### Study design and participants

The PlanHab Study took place at the Olympic Sport Centre Planica (Ratece, Slovenia) which is situated at an altitude of 940 m above sea level. In total, 65 healthy males were screened and 14 were included in the trial. Individuals with a recent exposition (<2 months) to altitudes above 2000 m and individuals normally residing at altitudes higher than 500 m were excluded. All participants gave written informed consent. 11 participants finished all three campaigns; 3 dropped out before the last campaign due to personal reasons. The participants’ demographical data were as follows: age 26.4 ± 5.2 years; body mass 75.9 ± 10.6 kg; stature 1.8 ± 0.05 m; BMI 23.5 ± 2.8 kg/m^2^ (mean ± SD).

The participants were exposed to three different protocols: normobaric normoxic horizontal bed rest (NBR: FiO_2_ = 0.209%; PiO_2_ = 133.1 ± 0.3 mmHg), normobaric hypoxic horizontal bed rest (HBR: FiO_2_ = 0.141 ± 0.004%; PiO_2_ = 90.0 ± 0.4 mmHg; equivalent to ∼4000 m) and normobaric hypoxic ambulatory confinement (HAMB: FiO_2_ = 0.141 ± 0.004%; PiO_2_ = 90.0 ± 0.4 mmHg). In three campaigns executed between October 2012 and October 2013 every participant consecutively passed each protocol (cross-over design). The campaigns were separated by an approximate 4-month wash-out period and each campaign lasted 32 days. In the first 7 days after the participants‘ arrival the baseline data was collected (BDC). During the next 21 days the participants were confined to the respective condition (HAMB, HBR or NBR) followed by a 4-day recovery period to obtain post-confinement measurements (R). Each campaign was entered sequentially in a fixed order by two participants per day.

Furthermore, the participants were subjected to a standardized diet that was strictly applied throughout each campaign.

### Bed rest and maintenance of hypoxic conditions

During bed rest conditions (HBR and NBR) the participants were strictly restricted to a horizontal position avoiding any strenuous physical activity. These restrictions implicated all daily routines (e.g. showering/toilet) though a change of position from lateral to supine or prone was allowed as well as passive stretching by a physiotherapist.

In contrast, when being assigned to the hypoxic ambulatory condition (HAMB), subjects were encouraged to maintain a level of physical exercise comparable to their usual daily routine by moving around in the restricted area (110 m^2^) and performing exercise sessions (e.g. cycling).

The circadian rhythm of all subjects was regulated by a standardized wake/sleep cycle (wake-up 7:00 am; lights off 11:00 pm). In the facility, environmental conditions were controlled and maintained at the same level through all campaigns (ambient temperature: 24.4 ± 0.7 °C; relative humidity: 53.5 ± 5.4%; ambient pressure: 684 ± 4 mmHg).

Hypoxic conditions were maintained with an O_2_ dilution system and have been reported in detail previously^7^. The maintenance of a sufficient O_2_ level during hypoxic conditions was surveyed continuously with portable O_2_ sensors (RAE PGM-1100, California, USA) with an alarm triggered at a pre-set O_2_ level of 13.5%.

### Blood sampling, processing and analysis

Blood collection took place 2 days before the beginning of the intervention period (baseline data collection, BDC), at days 2, 5, 14 and 21 during the confinement and 2 days after the end of confinement (R2). The blood was drawn from an antecubital vein from the fasted subjects in a horizontal, supine position at 7:30 in the morning.

For the assessment of hemolysis, the quantitative measurement of sICAM-1 and Zonulin, EDTA-anticoagulated blood was centrifuged at 3500 RPM for 5 minutes, the supernatants transferred into Eppendorf tubes and directly frozen at −80 °C. For the analysis, plasma samples were thawed in ice water and subsequently processed.

#### Purine analysis

A 5 ml syringe prefilled with 2 ml of an ice-cooled stop solution (composed of 6.3 mg EHNA, 7.446 g Na_2_EDTA, 7.608 g EGTA, 6.482 g D,L-α-glycerophosphate, 100 mg dipyridamole and NAOH to titrate a ph = 6 in 1 l of NaCl 0.9%) to prevent any supplementary formation or degradation of adenosine was used to draw venous blood samples. After transfer into a gel-vacutainer and centrifugation at 4000 RPM for 5 minutes, the samples were frozen in an upright position at −80 °C. Plasma concentrations of the purine nucleoside adenosine and inosine were analyzed by dual-column switching high-affinity performance/reversed-phase high performance liquid chromatography (HPLC, Chromosystem, Martinsried, Germany) as described previously^8,9^.

#### Assessment of hemolysis

Before assessing hemolysis in the subjects’ samples standard curves were established. Here, 2 ml of whole blood were centrifuged at 4000 RPM for 5 minutes, the supernatant discarded and the pellet relocated with 1 ml Aqua dest. and incubated for 1 hour at room temperature to induce hemolysis. After further centrifugation at 4000 RPM for 10 minutes, the supernatant was kept as 100% lysate and the pellet was discarded. A dilution series was prepared by serial dilution of the 100% lysate with Hanks buffered salt solution (HBSS) to acquire 2.5, 1.0, 0.125, 0.0625, 0.03125, 0.015625, 0.0078125 and 0.00390625% hemolysed samples. After centrifugation at 4000 RPM for 10 minutes the supernatants were measured with the Thermo Scientific^TM^ Nano Drop 2000/2000c Spectrophotometer (Erlangen, Germany) at 414 nm to establish a standard curve.

Subsequently, the plasma samples from EDTA-anticoagulated blood were thawed, mixed and 1 µl of the sample measured at 414 nm (Thermo Scientific^TM^ Nano Drop 2000/2000c Spectrophotometer, Erlangen, Germany). Its percentage of hemolysis was then detected via the established standard curve.

#### Erythropoietin analysis

Lithium-heparinized blood (2 ml) was transferred into a gel-vacutainer, centrifuged and frozen at −80 °C. Erythropoietin concentrations were determined by sandwich enzyme-linked immunoassay as described previously by Keramidas *et al*.^10^.

#### Cell blood count

Hemoglobin, hematocrit, mean corpuscular volume (MCV), erythrocytes, reticulocytes and thrombocytes were analyzed with an automated laser-based hematology analyzer (Advia 120; Siemens, Munich, Germany) within 8 h after blood sampling.

#### Body mass and composition

Body mass and composition were assessed daily in the supine position using a calibrated, custom-made gurney incorporating load cells (Sigma 6 C, Libela ELSI, Celje, Slovenia) and a fan-beam dual energy X-ray absorptiometer (DXA; Discovery W-QDR series, Hologic, Bedford USA) as described previously^7^.

#### Soluble intercellular adhesion molecule-1 (sICAM-1)

Quantitative measurement of sICAM-1 was carried out by using the commercially available abcam^®^ ICAM1 Human ELISA Kit (abcam^®^, Cambridge, MA, USA) according to the manufacturer’s guidelines. Plasma samples and a 1:10 dilution was used for the measurement.

#### Zonulin

Quantitative measurement of Zonulin was carried out by using the commercially available IDK^®^ Zonulin ELISA Kit (Immundiagnostik AG, Bensheim, Germany).

### Data availability

The datasets generated and/or analysed during the current study are available from the corresponding author upon request.

### Statistics

Data were analyzed and plotted with SPSS 23.0 (IBM, Armonk, NY) and Sigma Plot 12.5 (Systat Software Inc., San Jose, CA) as reported previously^11^. Outcome variables and residuals were tested for deviations from the normal distribution using Kolmogorov–Smirnov tests followed by a Box–Cox Transformation where residuals were not normally distributed. Statistical inferences regarding the effects of different conditions and time points during the same campaign were imputed through mixed linear models (LME). We included fix effects for campaign, sequence of campaigns, potential carryover from the previous campaign, condition, time within the respective campaign, and the interaction of condition and time. Random effects were included for subject and carry-over using a covariance matrix with variance components structure. A p value of <0.05 was regarded as statistically significant.

## Results

### Purine concentrations and kinetics

#### Adenosine

**Figure 1:**
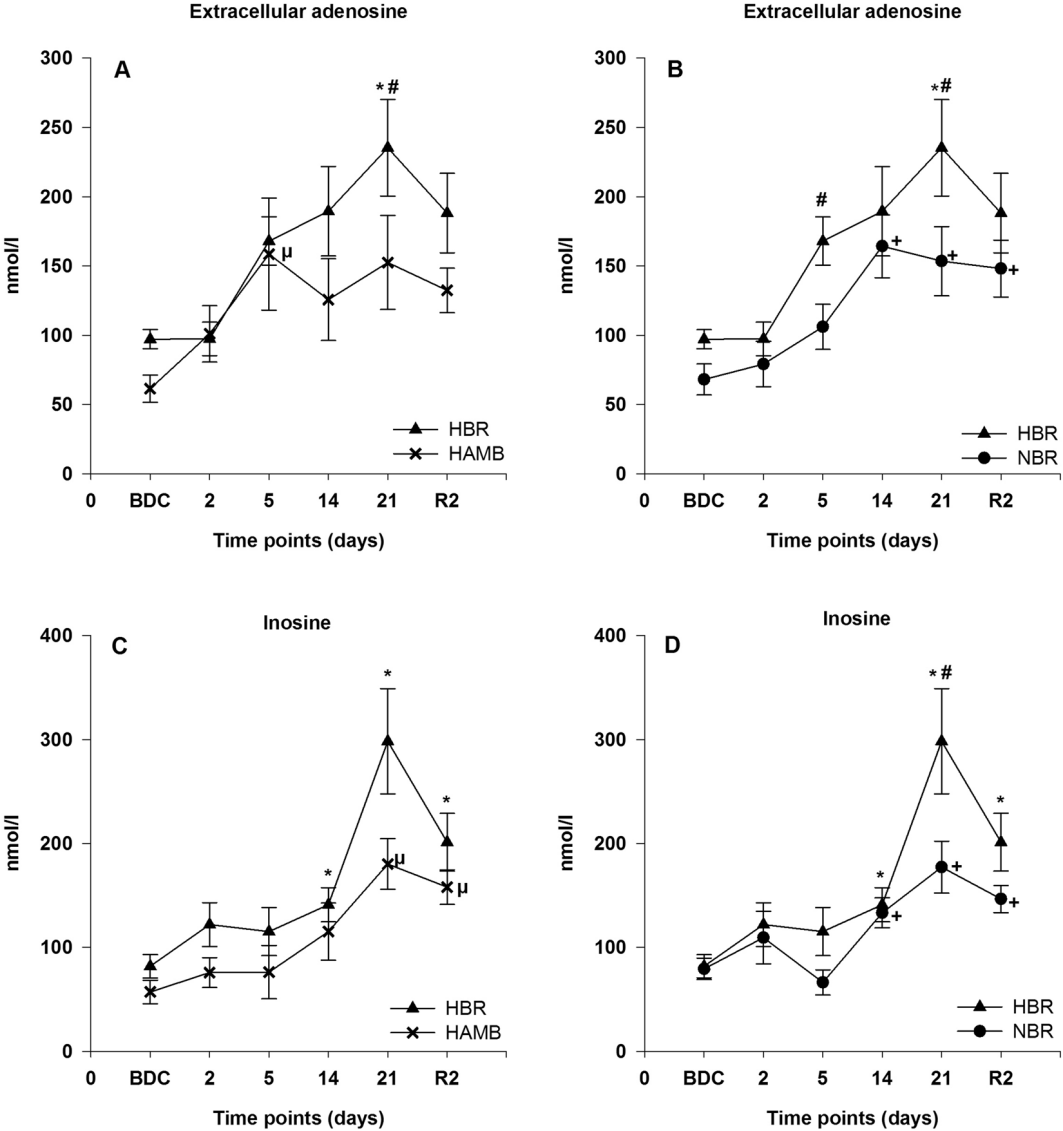
Extracellular adenosine (**A**,**B**) and inosine (**C**,**D**) concentrations in plasma; data are means ± SEM; units are nmol/l; HBR = hypoxic bed rest (n = 12–14); NBR = normoxic bed rest (n = 11–13); HAMB = hypoxic ambulation (n = 12); BDC = Baseline Data Collection; R2 = 2 days after the end of condition; ^**#**^Significant difference between HBR and HAMB or NBR; *Significant difference to BDC in HBR; ^µ^Significant difference to BDC in HAMB; ^**+**^Significant difference to BDC in NBR (p < 0.05).

The concentration of extracellular adenosine was significantly augmented during the intervention period in all three conditions, with a first peak on day 5, and a maximum value occurring mostly between days 14 and 21. The highest adenosine concentrations of over 200 nmol/l were observed in HBR with a high statistical significance to BDC at day 21 (p < 0.05 (in the comparison between HBR vs. HAMB) and p < 0.01 (in the comparison between HBR vs. NBR)). After the end of the interventions adenosine concentrations remained elevated in all three groups with a statistical significance at R2 in NBR compared to BDC (p < 0.05). A difference between the condition HBR and NBR was found at day 5 and 21 and between HBR and HAMB at day 21 (p < 0.05, respectively) (Fig. 1A,B).

#### Inosine

Inosine concentrations were significantly augmented during the intervention period in all three conditions, with a peak occurring at day 21. The highest inosine concentrations of ~300 nmol/l were attained in HBR at day 21. A significant difference was observed between HBR and NBR at day 21 (p < 0.05). During the recovery period, the inosine concentrations declined in all three interventions, but were sustained at a level which was significantly different compared to BDC at R2 (each intervention respectively p < 0.01) (Fig. 1C,D).

#### Assessment of hemolysis

**Figure 2:**
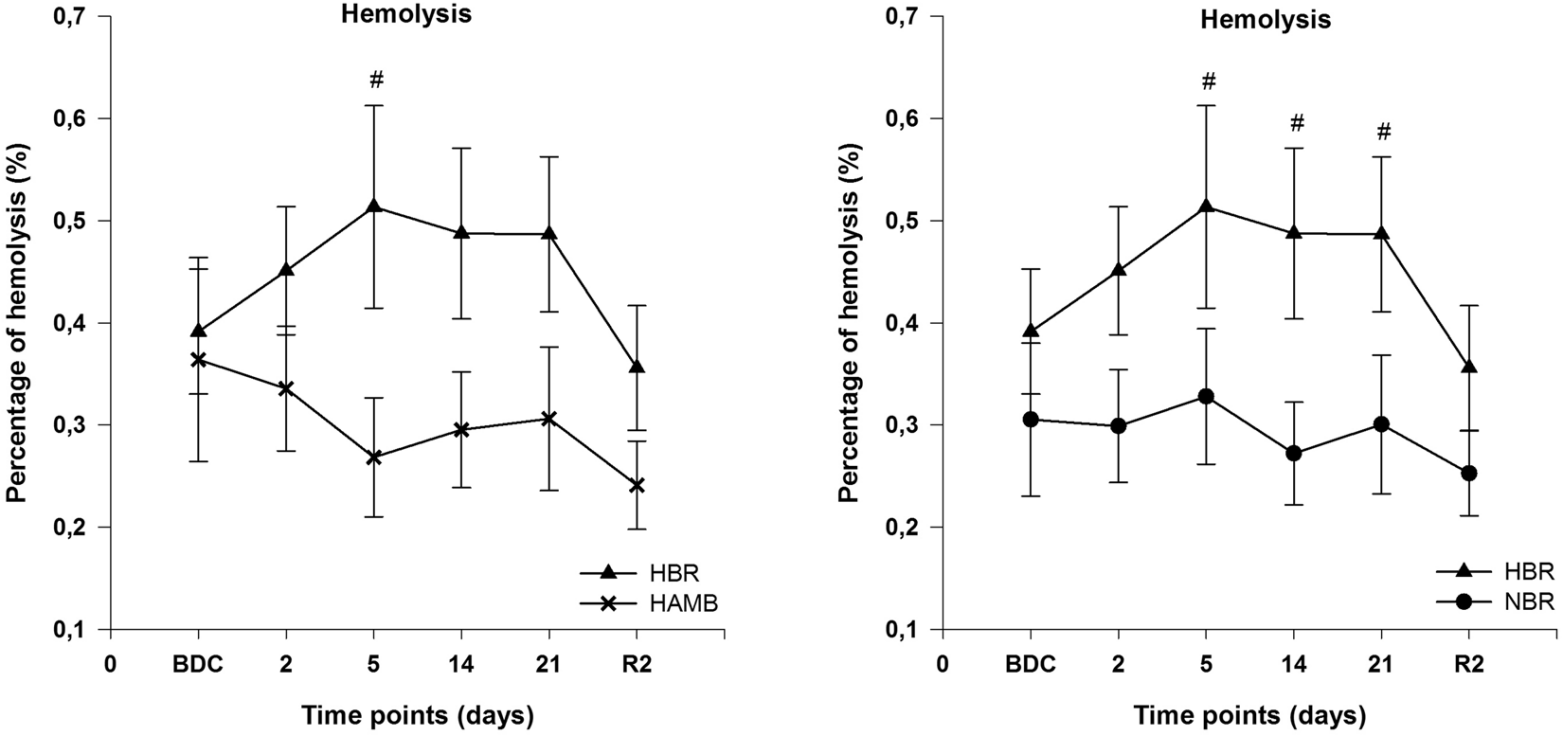
Assessment of hemolysis; data are means ± SEM; units are percent (%); HBR = hypoxic bed rest (n = 12–14); NBR = normoxic bed rest (n = 11–13); HAMB = hypoxic ambulation (n = 12); BDC = Baseline Data Collection; R2 = 2 days after the end of condition; ^**#**^Significant difference between HBR and HAMB or NBR (p < 0.05).

The percentage of hemolysis was significantly different between HBR and HAMB at day 5 (p = 0.041), and HBR and NBR at day 5, 14 and 21 (p = 0.044; p = 0.018; p = 0.031). In each intervention group no significant differences were detected versus BDC. In HAMB and NBR the percentage of hemolysis sustained on a constantly low level (Fig. 2).

#### Serum erythropoietin

**Figure 3:**
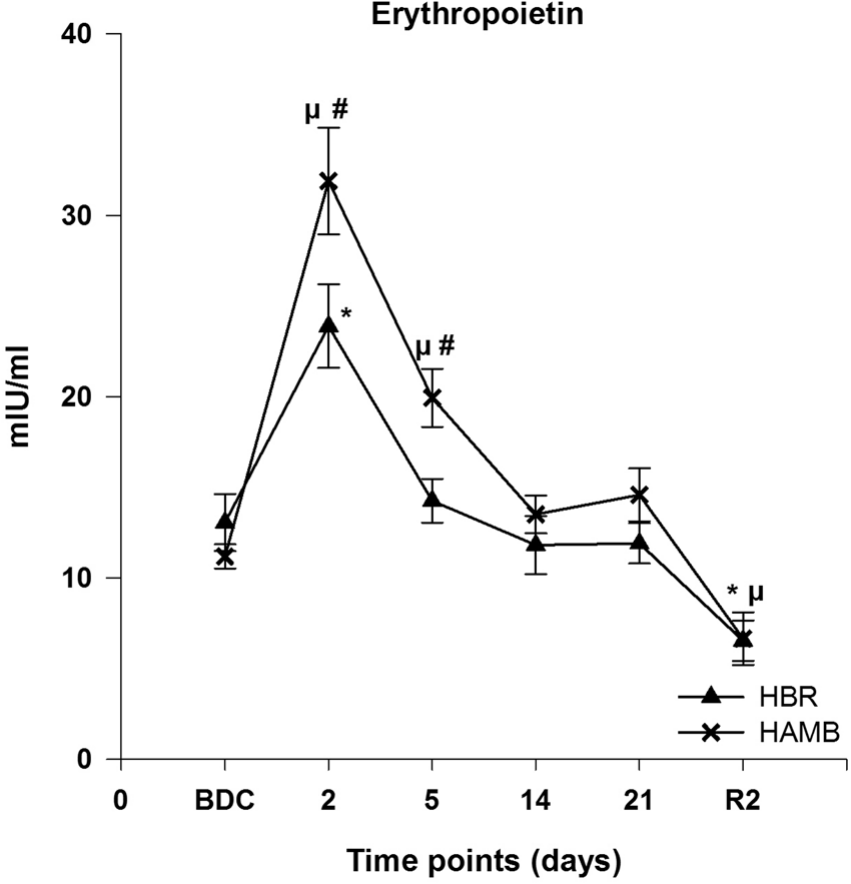
Absolute values of erythropoietin concentrations in serum; data are means ± SEM; units are mIU/ml; HBR = hypoxic bed rest (n = 11); HAMB = hypoxic ambulation (n = 11); BDC = Baseline Data Collection; R2 = 2 days after the end of condition; ^**#**^Significant difference between HBR and HAMB; *Significant difference to BDC in HBR; ^**µ**^Significant difference to BDC in HAMB (p < 0.05).

Highest erythropoietin concentrations were attained in the acute phase of exposition to HBR or HAMB at day 2, respectively, with a rapid decline to baseline or even lower values at the end of the intervention period and R2. A statistical significant difference to BDC was detected in HAMB at day 2 and 5 and in HBR at day 2 (p < 0.01 respectively). A significant difference between HAMB and HBR was detected at day 2 and 5 (p = 0.018 respectively) during the intervention period (Fig. 3).

#### Cell blood count

During the intervention periods all three conditions exhibited a significant hemoconcentration with elevated hematocrits. Hemoglobin was also augmented significantly during all conditions, whereas the mean corpuscular volume stayed nearly constant. A significant thrombocytosis was detected during HBR and HAMB (Table 1).

**Table 1:** Cellular blood parameters; data are means ± SD; MCV = mean corpuscular volume; HBR = hypoxic bed rest (n = 14); NBR = normoxic bed rest (n = 13); HAMB = hypoxic ambulation (n = 12); BDC = Baseline Data Collection; R2 = 2 days after the end of condition; ^#^Significant difference between HBR and NBR; ^+^Significant difference between HAMB and HBR; *Significant difference between HAMB and NBR at the respective time points; ^µ^Significant difference vs. BDC in the respective group (p < 0.05).

	Time points	HBR	NBR	HAMB
Hemoglobin (g/dl)	BDC	14.63 ± 0.75	14.60 ± 0.91	14.67 ± 0.56
	2	^µ^15.84 ± 0.95	^µ^15.83 ± 1.03	^µ^15.65 ± 0.59
	5	^µ^16.54 ± 1.06	^µ^15.80 ± 1.03	^µ^15.69 ± 0.83
	14^#,+^	^µ^17.50 ± 1.03	^µ^16.05 ± 1.13	^µ^16.12 ± 0.57
	21^#^	^µ^17.09 ± 1.13	^µ^15.72 ± 1.26	^µ^16.42 ± 0.83
	R2	15.33 ± 1.15	15.37 ± 2.04	15.05 ± 0.92
Hematocrit (%)	BDC	43.93 ± 2.12	44.15 ± 2.62	43.86 ± 2.21
	2	^µ^47.31 ± 2.55	^µ^47.92 ± 2.95	^µ^47.10 ± 1.72
	5	^µ^49.63 ± 2.97	^µ^47.49 ± 2.71	^µ^47.18 ± 3.09
	14^#,+^	^µ^51.11 ± 2.84	^µ^47.41 ± 3.21	^µ^48.09 ± 2.40
	21^#^	^µ^50.58 ± 3.31	46.42 ± 3.38	^µ^48.90 ± 2.96
	R2	44.91 ± 3.71	45.47 ± 6.31	44.98 ± 3.18
MCV (fl)	BDC	84.64 ± 4.66	85.99 ± 4.68	87.89 ± 3.48
	2	84.44 ± 5.10	85.99 ± 4.52	87.45 ± 3.50
	5	84.06 ± 4.62	85.39 ± 4.55	87.80 ± 3.36
	14^+^	^µ^82.36 ± 5.11	^µ^84.62 ± 3.84	87.31 ± 4.38
	21	83.51 ± 5.34	^µ^84.13 ± 3.81	87.40 ± 4.86
	R2	83.63 ± 4.97	^µ^84.11 ± 4.20	88.17 ± 4.65
Thrombocytes (G/l)	BDC	206.14 ± 76.65	197.92 ± 45.50	201.00 ± 42.36
	2	233.86 ± 68.12	204.77 ± 49.37	229.83 ± 49.13
	5	^µ^245.21 ± 51.08	215.08 ± 64.21	215.08 ± 56.22
	14	^µ^260.29 ± 66.18	221.08 ± 75.07	^µ^238.17 ± 40.30
	21	^µ^237.36 ± 82.19	200.00 ± 69.38	212.92 ± 46.74
	R2	210.14 ± 80.03	171.39 ± 73.27	196.58 ± 36.86

**Figure 4:**
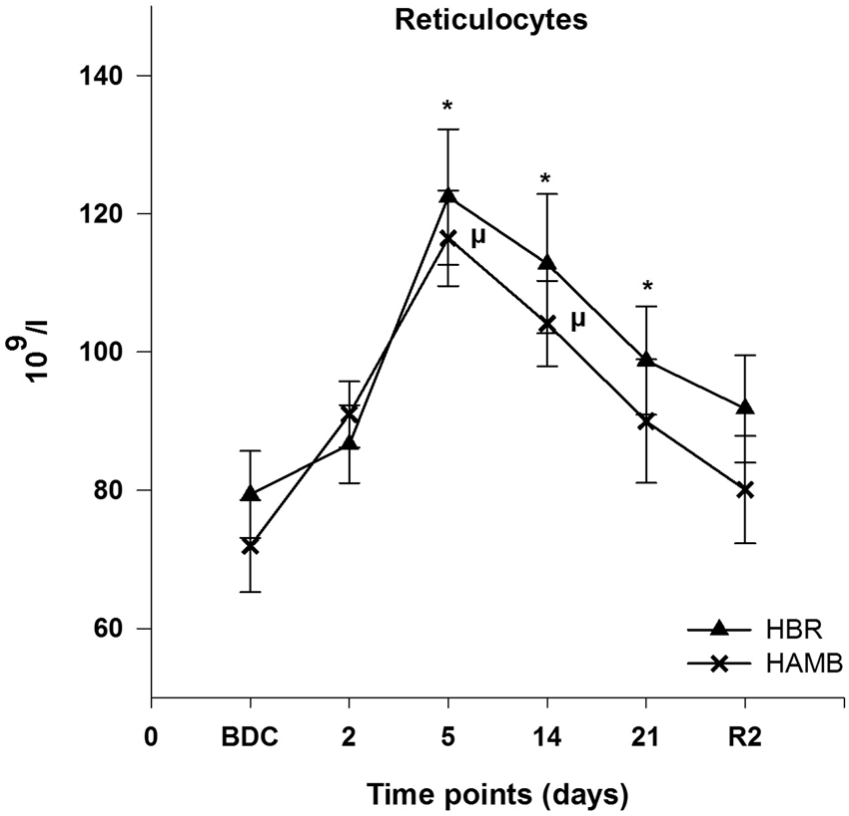
Reticulocyte count; data are means ± SEM; units are 10^9^/l; HBR = hypoxic bed rest (n = 11); HAMB = hypoxic ambulation (n = 11); BDC = Baseline Data Collection; R2 = 2 days after the end of condition; *Significant difference to BDC in HBR; ^µ^Significant difference to BDC in HAMB (p < 0.05).

Reticulocytes increased significantly during HBR and HAMB with their maximum peak at day 5 (Fig. 4).

#### Body mass and composition

Body mass and whole body fat free mass were significantly reduced in the 4-day recovery phase after each intervention compared to BDC. Data has been published previously^7^.

#### Soluble intercellular adhesion molecule-1 (sICAM-1)

No significant changes between the three interventions or between the intervention period and BDC in each intervention were detected (Table 2).

**Table 2:** sICAM-1 and zonulin; data are means ± SD; units are ng/ml; HBR = hypoxic bed rest (n = 6); NBR = normoxic bed rest (n = 6); HAMB = hypoxic ambulation (n = 6); BDC = Baseline Data Collection.

	Time points	HBR	NBR	HAMB
sICAM-1 (ng/ml)	BDC	100.75 ± 45.12	71.75 ± 10.12	85.57 ± 45.82
	2	105.65 ± 46.57	77.62 ± 6.20	86.05 ± 30.26
	14	101.28 ± 47.40	67.06 ± 15.13	84.27 ± 45.93
	21	114.62 ± 49.99	71.25 ± 13.59	74.56 ± 9.91
Zonulin (ng/ml)	BDC	10.99 ± 1.88	13.01 ± 8.56	13.39 ± 4.91
	2	14.55 ± 6.02	12.93 ± 2.95	20.01 ± 8.35
	14	10.91 ± 2.32	11.69 ± 2.77	10.50 ± 2.65
	21	10.85 ± 3.45	11.94 ± 4.51	14.86 ± 3.10

#### Zonulin

No significant changes between the three interventions or between the intervention period and BDC in each intervention were detected (Table 2).

## Discussion

The PlanHab study was designed to fulfill the highest possible level of standardization to examine in humans the adaptation of organ systems to defined environmental factors anticipated for human exploration missions. Two main environmental factors were implemented in PlanHab: hypoxia to verify effects of the anticipated atmospheric conditions, and bed rest as model to mimic reduced gravitational forces. Therefore, PlanHab and its high fidelity conditions offered, for the first time, the possibility to investigate the independent or combined effects of these extreme conditions on the kinetics of the purine adenosine and its metabolite inosine, both recognized as important markers of cell stress and metabolism especially as a function of hypoxia^3,6,12^. Hence, we hypothesized a hypoxia-triggered early-stage increment in their kinetics with increased adenosine and inosine concentrations.

This hypothesis was confirmed during PlanHab as purine concentrations increased in both hypoxic groups during the intervention period, but interestingly two unexpected findings were noted: i) bed rest seemed to boost adenosine release in combination with hypoxia as highest purine levels were attained during HBR, and ii) bed rest *per se* does seem to induce – although to a much lower degree - adenosine release since its concentrations were also augmented under normoxic conditions (NBR). To investigate and explain the hypoxic-enhanced release of adenosine, we further examined potential physiological mechanisms that may have contributed to these adenosine changes: we verified its potential source of release from red blood cells, from mal-perfused tissues, or as function of physical activity or nutritional changes.

Red blood cells are well known to store adenosin-5-triphosphate (ATP) to maintain energy supply and it was demonstrated that adenosine signaling plays an essential role in their metabolism as well as in their capacity to react to hypoxia^13–15^. ATP release from erythrocytes occurs in response to hypoxia and hypercapnia as well as from deformation^16,17^. Beside specific release mechanisms, hemolysis was found to be at its origin dependent on the oxygenation status of the cells^18^. Sikora *et al*.^19^ even stated that hemolysis seems a primary ATP-release mechanism in human erythrocytes as they observed that ATP release, independent of the stimulus, was exclusively triggered by hemolysis. That is why we quantified hemolysis during PlanHab and looked for its relation to other well-known hypoxia-triggered pathways as the erythropoietin- neo-erythrocytosis- axis. The glycoprotein hormone erythropoietin (EPO) represents an essential factor for the viability and proliferation of erythrocytic progenitors. Its release is known to be triggered by hypoxia via hypoxia-inducible transcription factors and induces a reticulocytosis. Significantly elevated EPO concentrations with a subsequent reticulocytosis were also observed in both hypoxic groups during PlanHab. However, this reticulocytosis resulted in a quantifiable hemolysis only during HBR and not HAMB. This was confirmed by a significant (p < 0.01) positive correlation for hemolysis and reticulocytes in the HBR group (data not shown). Apparently, only the combination of hypoxia with bed rest seems to be a sufficient trigger for a measurable hemolysis in this set-up and not hypoxia alone. To explain this phenomenon, we searched for mechanisms peculiar to bed rest. Feuerecker *et al*.^20^ suggested that bed rest modifies blood flow via fluid redistribution and thus causes shear forces that induce shedding of adhesion molecules. When these shear forces now encounter instead erythrocytes that were already primed by hypoxia in the sense that their deformability was decreased and their susceptibility to cell damage increased^21,22^, subsequent hemolysis potentially accounts for the extracellular adenosine increase. In this context, fluid redistribution under bed rest conditions might be one possible explanation for the combined effect of bed rest and hypoxia concerning hemolysis. Our findings during PlanHab support this hypothesis as fluid redistribution was most prominent in the HBR intervention^10^. Nevertheless, the question why the purine concentration was enhanced in all three interventions remains unresolved. During NBR and HAMB fluid redistribution and hemolysis were low, excluding these mechanisms as sole explanation. Against the background of the *in vitro* study results of Sikora *et al*.^19^, focusing on hemolysis as explanation, enhanced hemolysis should have been detected in each intervention to explain the increased adenosine concentrations. Thus, perhaps the degree of hemolysis could not linearly be quantified with the sensitivity of the analysis method of hemolysis, which is supported by the fact that a reticulocytosis was present also during HAMB and NBR (data not shown), but no subsequent enhanced hemolysis could be detected in these two interventions. However, a positive correlation for hemolysis and reticulocytes was observed in NBR (p < 0.01; data not shown) supporting this hypothesis. Additionally, further yet unidentified influences might be responsible for the stated adenosine release. Evidence exists that adenosine promotes the permeability of the blood-brain-barrier^23,24^ and that sustained adenosine exposure causes lung endothelial barrier dysfunction^25^. We hypothesized that a general mal-perfusion and subsequent hypoxic state in certain tissues is generated as a consequence of the constant ground reaction force-induced compression of dependent tissues during bed rest. Thus, an adenosine release is induced that impacts on the endothelial barrier function increasing its permeability, which is finally reflected in an increase of endothelial functional markers. Therefore, we quantified i) zonulin, a known physiological modulator of intercellular tight junctions that plays an important role in regulating intestinal permeability and is linked to the development of chronic inflammatory diseases^26–28^, and also ii) intercellular adhesion molecule-1 and its soluble form (sICAM-1), another functional endothelial marker present on endothelial cells that facilitates leukocyte adhesion and migration^29^ and is raised in inflammatory states^30,31^. Both markers have been implicated in a variety of pathological states (headache, typ-II-diabetes)^32,33^. However, despite the stated fluid redistribution that also hints to possible endothelial barrier dysfunction, we could not detect any increase in those markers which led to the rejection of this hypothesis to explain the observed adenosine release.

We further examined the effects of inactivity realized through bed rest on body composition and muscle mass. A battery of existent literature deals with the effect of physical training on muscle oxidative capacity, but conflicting results are demonstrated with either enhanced or reduced release of purines to plasma^34,35^. However, studies investigating the inverse state, physical inactivity, especially in humans are rather scarce as already stated by Gram *et al*.^36^. They seem to suggest the inactivity-induced down-regulation of the multiple purine-dependent pathways but query at the same time that these adaptions may not necessarily be opposite to that of physical training. Against this background, the analysis of our study colleagues Debevec *et al*.^7^ on the body composition of the PlanHab subjects provides evidence that muscle catabolism in this setting might be one possible explanation for the significant increase of purine concentrations. Body mass and whole body fat free mass were significantly reduced from BDC to recovery. Against what was hypothesized, hypoxia did not aggravate these findings. Interestingly, these results were found in all three interventions and not only in the bed rest groups. Debevec *et al*. suggested that this might be underlined appetite reduction and subsequent insufficient energy intake, low activity levels and/or effects of confinement *per se*.

In summary, our results confirm our initial hypothesis and clearly demonstrate that environmental hypoxia provokes a significant adenosine increase. Our analysis further suggests that this increase might be associated to neocytolysis. Unexpectedly, increased adenosine concentrations under normoxic conditions indicated that hypoxia-associated changes cannot be the sole explanation for our findings. However, other adenosine-related changes such as increased endothelial permeability could not be detected with the data given. In conclusion, our results can only partly be explained through hypoxic effects alone and other explanations remain to be provided. In this context, it has to be stressed that the one common factor of all three intervention groups in this study was the strictly controlled and standardized diet applied through all three campaigns. This might be one influential factor for the observations made.

### Limitations

The unexpected findings during the PlanHab study prompted us to verify different pathways to search for explanations. Unfortunately, due to restrictions in the volume and frequency of blood sampling, we were limited in the number of analyses we could conduct. A supplemental normobaric normoxic ambulatory confinement (NAMB) intervention would have been relevant in this cross-over designed study, but could not be realized due to financial limitations. Furthermore, the impact of nutrition could have been investigated by introducing a NAMB intervention with and without an applied diet.
